# On Unreduced Dislocations of the Shoulder

**Published:** 1875-10

**Authors:** Edward N. Brush


BUFFALO

Medical and Surgical Journal.

VOL XV. OCTOBER, 1875. No. 3.

### Original Communications.

:o:

ART. I.—*On Unreduced Dislocations of the Shoulder Joint (sub-glenoid) with the report of Two Cases, One of Two Hundred and Thirty Days Duration, and One, Congenital, of Eleven Months, Reduced by Prof. J. F. Miner, M. D., Together with reference to other Cases of long standing which have fallen under my Observation.* By EDWARD N. BRUSH, M. D.

Dislocations of the shoulder occur more frequently than of any other articulation, and the diagnostic signs of the luxation are such that the accident is recognized in most cases with but slight difficulty. It some times, however, occurs that owing to other complications, to great swelling, or absence of pain, that the accident is overlooked, or, if suspected, is not recognized. Owing also to the known liability of this joint to consecutive luxation, cases occur in which, after the dislocation has been properly reduced, re-luxation results, which is, owing to the absence, or superficial character of subsequent examinations, overlooked.

It thus occurs that a certain number of unreduced dislocations of this joint are constantly coming forward for advice and treatment. Sir Astley Cooper placed the period at which reduction could take place at three months, this seems, however, to be considerably within the limit of possibility, though the difficulties attending reduction rapidly increase as this period is passed.*

*Erichsen. Science and Art of Surgery, Vol. 1, page 413. Gross. A System of Surgery, Vol. II, page 76.

Thus reduction has been effected by Brodhurst after twenty-five weeks; by Nathan R. Smith after six, seven, eight, nine and ten months; by Malgaigne after eight months; by Caron du Pillard after six months, and by Sédillott after a year. By the use of subcutaneous division of the muscles and capsular ligament, Dieffenbach is said to have effected reduction at the end of two years.*

The interest which is naturally connected with a luxation of as frequent occurrence as that of the shoulder joint will admit of a brief resumé of the subject. First as to diagnosis. When seen soon after the accident this is generally easy to determine. The only accidents liable to be mistaken for it are fracture of the neck of the humerus, and epiphyseal fracture of the superior extremity of that bone.

A careful examination, the absence of the head of the bone from the axilla, and the presence of crepitus will generally suffice to distinguish fracture of the neck from dislocation. In epiphyseal fracture, however the case is different and the accident is frequently confounded with luxation. In separation of the epiphysis the crepitus, if obtained, is of a muffled character, resembling exactly that sometimes felt in old dislocations. In obedience also to a law which governs dislocations, the bones are moved into and retained in a definite place by the muscles. An intelligent appreciation of the symptoms of this fracture set forth by Prof. R. W. Smith in his treatise on fractures in the neighborhood of joints, and more recently by Dr. E. M. Moore,† of Rochester, will serve to distinguish it from dislocation; and the fact that it never occurs after

---

*Since Oct., 1872, the following cases have fallen under my observation:

CASE I. J——, aged 46, of C——, N. Y., dislocation into the axilla of twelve weeks duration treated as a sprain. Reduced by Dr. Miner under Ether on Oct. 23d.

CASE II. Eddie F———, aged eleven months. Dislocation since birth. This case will be referred to in detail in the course of the paper.

CASE III. J. L., of Oswego, N. Y., aged 32. On Sept. 14, 1873, he fell through a bridge, striking on his elbow. A physician was called who diagnosed fracture of the shaft of the humerus and applied splints. On the tenth of Dec. following, Dr. Miner, assisted by Dr. Cronyn, easily reduced the luxation by the use of extention and rotation of the arm. On Dec. 20th, patient was discharged well.

CASE IV. C. W. S., a blacksmith, aged 60, received an injury resulting in dislocation, Aug. 14, 1874. Unsuccessful efforts at reduction were made at his home in the country Having been sent to Buffalo, on the 24th of October, Dr Miner succeeded by means similar to those employed in other cases, namely extension, counter-extension and rotation in reducing the luxation. One month after the reduction, the arm, which had become somewhat atrophied, was found to be rapidly regaining its usefulness. (See report of clinical remarks. By W. W. Miner. BUFFALO MEDICAL JOURNAL, Dec., 1874, page 183, Case VIII.

CASE V Henry A——, aged 53. Dislocation of over seven months. See report in subsequent part of this paper

†Epiphyseal Fracture of the Superior Extremity of the Humerus. By E. M. Moore, M. D. Transactions of the American Medical Association, Vol. XXV, 1874.

the nineteenth or twentieth year, will exclude its consideration from many cases. In any case, however, the presence of dislocation may at once be determined by the application of a test first brought before the profession by Prof. Dugas,* of Georgia. When the head of the bone has left the glenoid cavity, if the hand of the dislocated arm be placed on the opposite shoulder, the elbow will project from the chest, and as long as the dislocation remains it will be found impossible to place the elbow against the chest with the hand in this position. Neither in a contusion nor a fracture is this the case.

Prof. Frank H. Hamilton has recently in a clinical lecture brought out two new differential signs in dislocations of the shoulder, which are fully set forth in the following extract from the lecture.†

"*First*, While the head of the humerus remains in its socket, if a rule be laid upon the outside of the arm from the shoulder to the elbow, it will not touch the acromion process, but will be distant from it at least half an inch, generally one inch or more. On the other hand, if the bone is removed from the socket, in whatever direction it may be displaced, whether forwards, downwards, or backwards, unless the shoulder is much swollen, the rule, placed in the manner above stated, will touch the acromion process.

*Second*, If, standing behind the patient (in case of the right shoulder) the thumb and forefinger of the left hand is made to grasp the top of the shoulder in such a manner as that the interdigital commissure shall rest upon the acromion process, just outside of the acromio-clavicular articulation; and if then the finger and thumb are dropped perpendicularly, the tip of the finger will (in case the head of the humerus is not dislocated) rest upon the center of the round upper extremity of the humerus, as it projects in front of the acromion process, while the end of the thumb will rest upon the head of the humerus behind; but the head will be felt indistinctly by the thumb, for the reason that, instead of projecting as it does in front, it actually recedes a little beneath the acromion process. Up to this moment the surgeon may entertain some doubt whether he is actually grasping with his thumb and finger the head of the bone; but if he now moves the elbow of the injured limb forwards, so as to carry the head of the humerus backwards in its socket, he will feel it press strongly upon the thumb, and this will be conclusive. If a dislocation exists, the

---

*Report on a New Principle of Diagnosis in Dislocations of the Shoulder Joint. By L. A. Dugas, M. D. Trans. Amer. Med. Assoc., Vol. X.

†The Medical Record, March 27, 1875, p. 220.

head of the bone cannot be felt in this situation, and by the thumb thus placed.

I have told you that both of these differential signs, in their application to shoulder joint injuries, are liable to one exception. The phenomena would be the same, so far as these two signs are concerned, whether there was a dislocation of the head of the humerus, or a fracture with displacement of the neck of the scapula. The latter accident must, therefore, be first excluded by a careful application of the rules of diagnosis given in our treatises upon surgery."

In a case which fell under my observation in January, 1875, Dr. J. F. Miner, by an application of Prof. Dugas' mode of diagnosis, and by obtaining crepitus, readily demonstrated that a case which had been treated for six weeks by a physician of experience in this city, for dislocation, was a fracture of the neck of the humerus.

Cases of so-called partial dislocation may make the matter of diagnosis obscure, but as it is the generally accepted belief of writers that this condition is only obtained as the result of a pathological and not of a traumatic cause, these may be excluded. When the long head of the biceps is displaced, or torn off, the head of the bone is carried upward and forward, producing what has doubtless been termed a partial dislocation by some surgeons. In a case of dislocation of the humerus, of ten weeks duration, reduced by Dr. Miner, on October 24, 1874, this condition was present; but as there was considerable deposit in the glenoid fossa, it is possible that the unnatural position of the bone was assumed for that reason, and that the biceps tendon was not torn. Should the dislocation of the shoulder be unrecognized through failure to apply the foregoing test or should attempts at reduction fail, or if successful, re-luxation occur and escape notice, what is the prognosis? As before stated, no period can be definitely stated at which reduction would not be possible. Prof. Gross tells us that he has failed in one instance at the sixth week, though he has succeeded at a much later period. It thus seems that a certain prognosis cannot be made, moreover such injury may be done to the circumflex nerve or axillary plexus that the arm, even if reduced, will be either wholly, or in part, paralyzed. This accident, however, may not be of a permanent character, slight injuries of the nerve being followed in a certain number of cases by recovery, under appropriate measures.

In no case should an unfavorable prognosis be made, where the nerve lesion is not plainly of a grave character, and where the time since the dislocation is not more than a year. When a new cavity has been formed for the head of the bone, or extensive inflammatory action has produced considerable adhesion, rendering the danger of tearing the axillary plexus, or artery, imminent, the propriety of an attempt at reduction may be questioned.

In attempting to restore an unreduced luxation of the shoulder joint, the surgeon has the choice of many procedures. As simple manual manipulation will, in all probability, prove futile, the first attempt should be made with the heel in the axilla. This simple method will often suffice, and is much better than the resort to the more complicated, and perhaps more dangerous, one of the use of the pulleys, or adjuster.

The patient may be placed on the floor on a mattress, or what is still better, upon the floor itself. Complete relaxation being produced with ether, the surgeon is to employ the various methods described for the reduction of the dislocation. It will be found in most cases that the head of the bone goes back into the glenoid cavity without the noise which accompanies the reduction of recent luxations. In fact, the surgeon will often be surprised to find on examination, that reduction has been affected without his notice. The reason of this probably lies in the fact that the glenoid cavity has become filled in by a deposit of new material. Should reduction be found to be impossible by any of the ordinary means, the tendons of the muscles surrounding the joint, and the capsular ligaments may be divided subcutaneously as has been practiced by Dieffenbach. In a case of twenty-five days standing, in Guy's Hospital, reduction was made by placing an air pad in the axilla and firmly binding the arm to the side. On removing the bandage at the end of three days the arm was found reduced.* Other methods will, however, prove as effectual as this, and certainly less tedious.

The accidents which may occur in attempts to reduce an old dislocation of the humerus are, fracture of the arm, rupture of some portions of the axillary plexus, rupture of the axillary artery

*Bryant. Diseases and Injuries of the Joints. 1859, p. 227.

or of the vein, and fracture of one or more ribs. Fracture of the humerus, especially if near the superior extremity, will, of course, render the reduction of the dislocation still more difficult, but the attempt should not be abandoned, even on this account. Dr. Hamilton cites an instance in which he succeeded in reducing a dislocation complicated by fracture, on the eighth day.* In September, 1873, J. M——, a pedler, residing on Michigan street, tripped on an irregularity in the walk, while carrying his pack, fracturing the humerus at the upper extremity. When brought to Dr. Miner's office, crepitus could be obtained on motion of the fragments, but there existed also a depression immediately beneath the acromion process, which indicated dislocation. Examination revealed the presence of the head of the bone in the axilla. Ether was administered, and by extension, aided by the fingers of an assistant to guide the head into the glenoid cavity, reduction was effected. This being accomplished, the fracture, which could now be plainly made out, was dressed, and at the earliest moment passive motion of the joint employed. The result was perfect union without impaired function of the arm.

Should it be found impossible to reduce the head of the bone, the fracture should be dressed and allowed to unite, and at the earliest moment possible another effort should be made to reduce the dislocation.

Rupture of the axillary artery or vein, or some portion of the axillary plexus is more apt to occur where extensive inflammatory adhesion has bound them to the surrounding parts. I am indebted to Dr. De Forrest Willard of Philadelphia, for the following list of cases in which rupture of either the artery or vein has taken place.†

"After a somewhat extended search through the reports of the hospitals of America and England, and in the various journals published in these countries, I have succeeded in collecting the following cases: The most complete records have been found in Malgaigne's Traitè des Fractures et des Luxations, and in Flaubert's Mèmoires sur plusieurs Cas de Luxations, etc., Rèpertoire d'Anatomie et de Physiologie, 1827, and Hamilton's Treatise on Fractures and Dislocations, 1871.

---

*Hamilton. Fractures and Dislocations. 1875, p. 566.

†Philadelphia Medical Times, Aug. 16, 1873, p. 721. Notes on Clinical Lecture by Prof. D. Hayes Agnew, M. D.

The sole case of laceration of the axillary vein alone is that mentioned by Froriep,*—a scrofulous women, æt. 36, in whom the dislocation had existed for twenty days. The vein was torn entirely across, and the patient died in one and a half hours.

This, and the one of Agnew's above recorded, constitute the entire list of accidents to the vein alone.

Platner† has put on record a case in which both vein and artery were injured, the patient dying of hemorrhage from the subsequent rupture of the sac.

Guérin‡ tore the entire arm from the body of a woman 63 years of age, in reducing a luxation of three months' standing, and of course all the structure at the axilla must have been severed.

Charles Bell§ also reports that during the use of the ambe at the Newcastle Infirmary not only was the axillary artery ruptured, but also the surrounding structures to such an extent that immediate amputation became necessary. It is highly probable that the vein was also injured; but the report is incomplete, especially in the fact that the result is not stated.

In Leudet's‖ case, in Rouen, in 1824, an enormous swelling appeared in the axilla, immediately after reduction, which, though imparting no pulsation to the fingers, could be seen with the eye to rise and fall with the contractions of the heart. Gangrene supervened in three days, and the patient died in ten, of hemorrhage. A post mortem examination revealed the fact that the artery was torn across, the rim of the glenoid fractured, and the muscles lacerated.

In Johnson's Medico-Chirurgical Review¶ are recorded several cases from the French of Flaubert,** in which that author states that he himself saw four cases of injury to the axillary or brachial vessels or nerves,and believes either that he has been singularly unfortunate or that other surgeons have not been honest in their reports.

One of these cases was an axillary dislocation, attempts at the reduction of which were followed by an enormous swelling, syncope, and extreme prostration, the patient narrowly escaping. Another was a dislocation of the elbow, which was followed by a similar result, the brachial artery probably having been injured. Another was undoubtedly emphysematous, as the tumor appeared during violent outcries, and was supra-clavicular, extending over the shoulder and to the back; and in the remaining case the nerves were lacerated. He also reports another case in which the patient died after attempts at reduction of a dislocated hip.

Verduc†† ruptured the artery, and the patient died.

---

*Veraltete Luxationen, etc., Weimar, 1834, p. 35.
†Hamilton on Fractures and Dislocations, 1871, p. 564.
‡Erichsen's Surgery, fifth American edition, 1869, p. 307.
§System of Operative Surgery, vol. ii. p. 246.
‖Gibson's Surgery, 1835, vol. i. p. 346.
¶Vol. xi. p. 452.
**Memoires sur plusieurs Cas de Luxations,etc., Repertoire d'Anat. et de Phys., 1827, obs. 3.
††Operations de la Chirurgie, 1693, t. i. p. 559.

Petit* and Delpech† met with similar accidents and similar results.

David,‡ Hôtel de Rouen, reduced a luxation of several weeks' standing, using immense force, and the patient died of mortification, the supposed cause being a rupture of the artery.

Pelletan,§ while attempting to reduce a luxation of four months' standing, discovered a tumor which he supposed to be emphysematous, but upon opening it the patient perished from hemorrhage.

Dupuytren‖ reports a case in which a woman 60 years of age had an axillary dislocation of six weeks' standing successfully reduced. Two or three months subsequently, however, a tumor appeared in the axilla, which, being mistaken for an abscess, was opened, and the patient died on the eighth day from secondary hemorrhage.

Nélaton¶ also had an aneurismal tumor appear three months after the reduction of an axillary dislocation, for the cure of which he tied the subclavian, but with what result is not recorded.

Malgaigne** was compelled to desist from attempts to reduce a displaced humerus of sixty-eight days' duration on account of the occurrence of a tumor in the axilla, which, being, however, subsequently arrested in its growth and absorbed under the use of ice, compression, etc , he decided to be due to rupture of the muscular branches instead of the axillary.

Desault†† met with two or more cases;‡‡ but they are to be received with some doubt, having been considered by him as "tumeurs aèriennes," or instances of emphysema. Malgaigne, however, believes that they were produced by a rupture of at least the muscular branches of the artery, "the pulse of the patient being scarcely perceptible in the side affected and a syncope which supervened appearing to favor the suspicion of a rupture; but, in the absence of fluctuation, of pulsation, and of any change in the color of the skin—the return of the pulse, the circumscription of the tumor, its resistance, and the sound caused by striking upon it, produced the belief that it was owing not to an effusion of blood, but to a disengagement of air that had been confined in the lacerated cells of the cellular membrane." The tumor entirely disappeared in thirteen days, leaving a large ecchymosis, however, for a month or more.

Callender§§ tore a small opening in the upper wall of the artery in attempting to reduce a dislocation of six weeks' standing. He

---

*Hamilton, *op. cit.*, p. 564,

†Malgaigne, Traite des Fractures et des Lnxations, 1855, t. ii. p. 152.

‡Amer. Jour. Med. Sci., 1828, p. 136, and First Lines of Pract. Surg., S. Cooper, vol. ii., p. 466.

§Rep Gen. d'Anat. et de Phys. Path. et de Chirurg. Clinique, 1827, t. ii. p. 95.

‖Lecons Orales, second edition, t. iii. p. 12; Pelletan, *op. cit.* p. 83.

¶Erichsen, *op. cit.*, p. 307.

**Desault's Works by Caldwell, p. 149; Journal de Chirurg., t. iv. p. 301.

††*Op. cit.*, p, 150.

‡‡Bichat's [illegible]emoire sur la Luxation de l'Humerus, in § 74 says occasionally, but in § 68 twice.

§§St. Barthol. Hosp Rep., 1866, vol. ii p. 96.

very wisely laid open the tumor, turned out the clot, and tied the vessel both above and below the bleeding orifice. The patient died soon after with symptoms of pulmonary embolism.

Warren,* of Boston, reports a case in which a man 30 years of age fell and dislocated his shoulder. Violent attempts at reduction were made, and, according to the statement of the patient, the booted foot of the operator was placed in the axilla. When he entered the hospital, the arm and shoulder were greatly swollen, but after this subsided the bone was found in its normal place. Five days subsequently, during a fit of coughing, something "gave way," soon followed by a large swelling and discoloration in the axilla. No pulse could be discovered at the left wrist, and the tumor in the axilla became enormous. In six weeks an abscess formed and opened, discharging dark, coagulated blood. Upon the third day there was a sudden gush of blood, which was arrested by pressure, and on the following day Warren tied the subclavian artery. The patient made good recovery, and in one year afterwards slight pulsations could be distinguished in the radial. In sixteen months the man was greatly improved.

Gibson† twice met with the accident, and was honest, as usual, in reporting each case. The first was in the person of a man 50 years of age, whose left shoulder had been dislocated for two months. For two weeks subsequent to the accident it had been treated as a fracture, and after this ineffectual attempts had been made to reduce the deformity. He was admitted to the Philadelphia Hospital, and, after various manipulations and the trial of all the ordinary means employed, success was at last attained, and the head of the bone slipped into its place. In a short time, however, a swelling was noticed in the axilla, which slowly increased in size, the patient being painless and cheerful, until the evening, when the symptoms of depression becoming alarming, Prof. Gibson was sent for, but arrived too late to save the patient.

At the autopsy the artery was found entirely torn across, it having been firmly bound down by old adhesions to the capsule of the joint where it surrounded the neck of the bone.

His second case was a man 35 years of age, who had dislocated his shoulder nine weeks previous to his entrance into the hospital, and who had already passed through four attempts at reduction, in one instance having been suspended by the axilla over a door.

After a week's depletion, in accordance with the practice in vogue at that time, the attempt was again made by Gibson, assisted by a number of prominent surgeons. Extension and counter-extension were maintained by pulleys for half an hour continuously, after which rotary movements and all the usual methods of

*Amer. Jour. Med. Sciences, vol. xi., N. S., 1846; Med.-Chirurg Transactions, vol. xxix., p. 25, 1846.

†Gibson's Surgery, vol. i., pp. 325, 334; Chapman's Philadelphia Jour. Med. and Phys. Sciences, 1823, vol. vii., p. 81; Amer. Jour. Med. Sciences, 1828, p. 136.

reduction were faithfully but cautiously and gently tried for one and three-quarter hours, when they were finally successful.

Upon the next day, however, a pulsating tumor was discovered in the axilla, and on the following morning Gibson proceeded to tie the subclavian. The man, however, sank and died on the eighth day.

At the post mortem examination, which was carefully and accurately made, and which is fully described in his report of the case, the left hand and forearm were found in a state of incipient gangrene, while the ligation wound showed no attempts at union.

The artery was firmly attached to the bone and articular capsule by a ligamentous substance so compact and with so short a portion of the artery between this point of adhesion and the false attachment to the rib that it could hardly have escaped injury. The ends were separated half an inch, the utmost limit allowed by the adhering bands. The external coat of the artery formed the sac of the aneurism; but this had ruptured and allowed the contents to escape in the connective tissue.

"The walls of the true aneurismal sac were so compact a texture, and its boundaries so well defined, that the conjecture of its having existed previously to the reduction of the bone and that its rupture was a distant and subsequent event, is rendered probable."

The great tuberosity of the humerus had been fractured, but had partially united.

Blackman,* of Cincinnati, attempted the reduction of a dislocation of sixteen weeks' standing, which had previously resisted the efforts of six men with pulleys for two and a half hours. The manipulations of reduction, rotation, abduction, and elevation had been continued about ten minutes, when a sudden tumefaction occurred in the pectoral region and rapidly increased in size.

No pulse could be felt at the wrist; and, believing the artery to have been ruptured, the axillary was tied at the upper third of its course. Pressure was made by a broad band and compressed sponge, and on the following day the bulk was greatly diminished.

The patient did well for several days, but on the eleventh he perished from a hemorrhage at the seat of the ligature.

Lister† recently attempted the reduction of a dislocated shoulder of eight weeks' standing. The displacement was of the subcoracoid variety; and while pulling upward, as in La Mothe's method, he heard a distinct crack. No swelling appearing, however, and the head of the bone being still in its false position, various manipulations were employed, and the pulleys used, traction being made both downward, outward and upward. The force employed was not excessive, and efforts were finally discontinued.

Almost immediately afterwards, however, his attention was directed to an enormous swelling which appeared below and behind

---

*Western Lancet, Nov., 1856.

†Lancet, Feb. 1, 1873; Edinburgh Medical Journal, March, 1873.

the axilla, almost as large as an adult human head. The pulse being absent at the radial of the injured side, a rupture of the axillary artery was diagnosed, and the operation of tying it commenced. An incision was made directly into the axilla, and the extravasated partially coagulated blood turned out. The arm being held off from the side, the artery was so compressed against the head of the bone that no hemorrhage occurred; but upon being relaxed by the side of the body a free bleeding took place. The orifice was at last found to be concealed upon the posterior wall, from which a stream was so poured out into a cavity and reflected forward that it seemed to be a regurgitant stream from a large branch like the subclavian. Careful examination, however, proving that the main vessel was ruptured, a ligature was applied both above and below the opening, the vessel cut across, and the ends again secured. The opening was found to be about two lines in diameter.

The head of the bone was then removed, to facilitate subsequent reduction. The patient, although greatly reduced by the loss of blood, rallied a little, but soon sank, and died in three hours.

Mr. Lister, in his report of the case, regrets that he did not transfuse.

At the autopsy the explanation of the accident was evident, and is so instructive that I quote it in full:

"The surface upon which the head of the bone had rested in its new situation simulated cartilage in smoothness and firmness, and was formed anteriorly by a dense fibrous structure, strengthened with a considerable amount of osseous deposit in the form of a spicula, proceeding chiefly, though not exclusively, from the coracoid process and the surgical neck of the humerus; a state of things fully confirming the wife's report as to the long time that had elapsed since the accident. The broad and strong osteo-fibrous band thus connecting the humerus with the coracoid process had lain over the head of the bone, constituting a partial capsule for the new joint. At the same time it was intimately connected throughout, by condensed tissue, with the sheath of the axillary artery, which lay over it. Thus the vessel, instead of being surrounded by loose and yielding structures, as in the natural state, was attached, through the medium of the osteo-fibrous band, to the coracoid on the one hand and the neck of the humerus on the other; and when these bony points were separated from one another by the lever-like action of the humerus when the limb was drawn upwards, the artery as well as the band was necessarily subjected to violent traction. Accordingly, the band, as strong as it was, was found to have been torn right accross, and the rent in it was exactly opposite to the rupture in the artery. The giving way of the vessel had been rendered the more easy by atheromatous degeneration of its walls, which was present in an advanced degree.

The vein also showed evidence of having been extremely stretched, by considerable narrowing of its calibre at the same situation.

"The chief lesson to be learned from this sad case seems to me to be that in subcoracoid luxations there may be, as early as eight weeks after the accident, such adhesion of the artery to the scapula and the humerus as to make it dangerous to employ even the manipulative treatment without the pulleys, and especially the upward traction of the limb. For, as was remarked before, and will now be readily understood, the snap heard in the preliminary manipulation was in all probability caused by the rupture of the fibrous band above described, accompanied by the giving away of the vessel."

In common with Mr. Lister, Prof. Agnew also believes that the method of reduction by traction upward is, in old dislocations, more liable to produce a laceration of the vessel; and although it has been his favorite mode, and will be continued in all recent cases, yet in those of long standing he will first attempt others which do not put the artery so greatly upon the stretch.

He also believes that the limitation of the circulation by controlling of the subclavian assisted greatly in keeping a sufficient supply of blood in the brain to prevent fatal syncope, and in such sudden cases compression even of the abdominal aorta would be beneficial in keeping up the proper meningeal supply.

Another case, which would come very properly under a report of this kind, is recorded by Adams.*

The rupture of the artery in this case was not produced by the surgeon, but by the same accident which occasioned the luxation. The patient was thrown down by a runaway horse, and was brought to the Jervis Street Hospital with his left humerus dislocated and with an enormous swelling of the axilla. No pulse could be detected in the radial or brachial arteries of the injured side, and the patient was cold and prostrated, and so relaxed that the bone was slipped into its place by a slight manipulation.

The deep axillary swelling remained stationary for several days, no pulsation being perceptible either in it or in the arteries of the limb. After ten days, O'Reilly found the tumor upon the increase, and tied the subclavian in the third portion. The patient was seized with erysipelas and lost two fingers by gangrene, but he finally recovered, and was under observation several years afterwards.

The above constitute all the cases which have been discovered, and I believe that the report is complete to the present time.

*Recapitulation.*—Rupture of vein alone, 2 cases,—Froriep and Agnew: 1 fatal, 1 recovered.

Rupture of vein and artery, 3 cases,—Platner, Guérin, Bell: 2 fatal, 1 not stated.

*Todd's Cycl. Anat. and Surg., art. on Shoulder, Abnormal Conditions of, p. 616; Holme's Surgery, vol. ii., p. 827.

Rupture of the artery, 19 cases,—Leudet, Flaubert, Verduc, Petit, Delpech, David, Pelletan, Dupuytren, Nélaton, Malgaigne, Desault (2 cases), Callender, Warren, Gibson (2 cases), Blackman, Lister and Adams: 12 cases fatal, 6 recovered, 1 not stated.

The axillary artery was tied in 3 cases,—Blackman, Callender, Lister: all fatal.

The subclavian was ligatured 4 times,—Gibson (fatal), Warren (successful), Adams (successful), and Nélaton (not stated).

(Gibson did *not* tie the subclavian in his first case, as reported in most of the Surgeries.)

Total, 24 cases: 15 terminated fatally, 2 are uncertain, and 7 recovered.

The successful cases were those of Desault (2), Flaubert, Malgaigne, Warren, Adams and Agnew.

The case of Adams perhaps ought not to be reckoned with the others; yet it is so parallel an accident that I have deemed it advisable thus to place it.

Serious injury has also frequently been done to the axillray nerves; in some cases even the plexus being torn from the spinal column, in others various degrees of paralysis of the arm having followed. Malgaigne, Hamilton, Lenoir, Larrey,* and others report a number of cases.

Michaux (Hôpital de Louvain, 1841)† in reducing a luxation of the elbow tore off the median nerve and the brachial artery. He amputated, and saved his patient.

Various other accidents are reported as the results of efforts at reduction, but the above are all that are directly connected with the dislocation under discussion.

The cases are certainly sufficiently numerous to warn us against employing too much force; yet in the immediate case under notice only manual traction was made."

In connection with the foregoing, the history of the following case may not prove uninteresting, and its relation may add something to our knowledge of the possibility of the reduction of old luxations of the shoulder joint:

On the twelfth of January, 1875, Henry A——, of Fredonia, Chautauqua Co., N. Y., a farmer, aged fifty-three, weighing two hundred and thirty pounds, a strong, muscular man, while lifting a hog, which he had just killed, by the "gambrel," and with his arms extended above his head, fell, injuring his right shoulder. A physician was called, who at once diagnosed the character of the injury, and proceeded to reduce the dislocation of the shoulder, by

*Bul. de la Soc, Chirurg., i. I.
†Debruyn, Des Luxations du Coude, These Inaug., Louvain, 1843, p. 77.

manipulation. The patient complained, however, of constant numbness in the hand and arm, stiffness, and inability to move the joint, a condition of things which time did not remedy.

On the thirtieth of August last Mr. A—— came to Buffalo to consult Dr. J. F. Miner. An examination of the shoulder at the the time showed that the dislocation was unreduced.

A room was obtained for Mr. A—— at the General Hospital, and on the following day, two hundred and thirty days from the receipt of the injury, Dr. Miner proceeded to reduce the luxation. The patient was placed on a mattress on the floor, and ether administered by Dr. Rochester until complete relaxation was produced. A sheet was passed around the body under the axilla, to be used as a counter-extending band; this was placed in charge of an assistant. Another sheet was folded, and fastened by a clove-hitch to the arm just above the elbow. Removing his boot, and placing his heel in the axilla, Dr. Miner began to manipulate the arm in order to break up the adhesions which had formed. Then, flexing the forearm upon the arm, and while extension was being made by an assistant, by means of the sheet, Dr. Miner grasped the arm and forcibly rotated it outward by means of the forearm. This failed to reduce the dislocation. Keeping up the extension, the arm was now carried outward nearly at right angles with the body, and the same movement again instituted. After repeating this two or three times, the bone was felt to glide into its socket, the new adhesion being plainly felt to give way, but without that audible "snap" which so frequently accompanies the reduction of recent cases. It was now found that the arm could be freely moved in all directions, and that with the hand on the opposite shoulder the elbow rested on the thorax. With every evidence of perfect restoration, the arm was firmly bandaged to the side, and the patient placed in bed.

Five days after the reduction the patient returned to his home, being able at that time to make considerable motion with the arm. The numbness and pain had almost wholly disappeared.*

Congenital Dislocations.—Dislocations of the shoulder joint,

*A letter from Mr. A——, dated October 15, says: "My arm is improving in motion and strength every day. I have no trouble with it slipping out of place."

present from birth, whether the result of violent expulsive action on the part of the uterus, of falls or blows received by the mother during pregnancy, of some pathological change incident to intra-uterine life, or of violence at the hands of the accoucheur, may for convenience be classed under this one head.

Guérin* ascribes to some form of muscular contraction, and to corresponding muscular paralysis, not only dislocations of the long bones but also clubfoot and torticollis. He affirms that he has established, incontestably, the dependence of this abnormal state of the muscular system, upon the absence of some portion of the central nervous system. Breschet and Delpech, two French authors, who have given this subject much study, hold similar views, especially in relation to clubfoot.

Dupuytren holds that the causes of dislocations arise with the earliest development of the fœtus.

Carnochan† says that it appears most in accordance with science, to refer muscular contraction, upon which congenital dislocations depend, to a perverted condition of the excito-motor apparatus of the medulla spinalis. Hamilton arranges all these views under three heads:‡

"First, the physiological doctrines, according to which congenital dislocations are due to an original defect in the germ, or to an arrest of development.

"Second, the pathological doctrines, which refer them to some supposed lesion of the nervous centres, to contraction or paralysis of the muscles, to a laxity of the ligaments, to hydrarthrosis, or to some other diseased condition of the articulating apparatus.

"Third, the mechanical doctrines, which recognize no intra-uterine dislocations, except those which are strictly traumatic. The causes being understood to be the peculiar position of the fœtus in utero, violent contractions or the constant pressure of the walls of the uterus, falls and blows upon the abdomen, and unskillful manipulation of the child in delivery."

Without, at the present time, entering into a discussion of these

*Guerin, Recherches sur les Luxations Congenitales; par Jules Guerin, Paris, 1841.

†A Treatise on the Etiology, Pathology and Treatment of Congenital Dislocations of the Head of the Femur; by John Murray Carnochan, M. D New York: 1850.

‡A practical Treatise on Fractures and Dislocations; by Frank H. Hamilton, M. D. 1871, p. 761.

various causes, it may be admitted that all are capable of producing the accident under consideration.

As the result of paralysis, or of some other pathological cause incident to intra-uterine life, it is probable that luxations of the shoulder will not assume any one of the three positions known to be caused by traumatic influences, without greater or less modification. With accidents due to violence done the mother during the latter periods of utero-gestation, or to the fœtus during delivery, it is different, and any of the three luxations may be the result. The diagnosis of dislocation of the shoulder joint in the infant, is to be made by the application of the same principles as in the adult. Attention will be called to it by an abnormal rigidity of the arm, a deviation of the humerus from its natural axis, and the evidence of pain manifested by the child when the limb is moved. The prognosis in cases seen immediately after delivery, where the luxation was caused by some accident incident thereto, is more favorable than where there exists some lack of development in the parts, or where the accident has happened early in intra-uterine life. When this dislocation is not discovered, or is left unreduced, a new articular surface may be formed for the head of the bone, as in the the following case of a lunatic, who died at the age of forty-two, having a congenital dislocation of both shoulders backwards. At the autopsy, Prof. R. W. Smith found the following condition: "The coracoid process was remarkably prominent, but the acromion was not so prominent as in accidental dislocations of the shoulder. The head of the humerus could be felt and seen, distinctly, moving with the shaft, upon the dorsal surface of the scapula. On removing the integuments, muscles, etc., no trace of the glenoid cavity could be found in the natural position; but upon the external surface of the neck of the scapula was a well-formed socket, which received the head of the humerus. This socket was covered with a cartilage of incrustation, and surrounded by a perfect capsule. The tendon of the biceps arose from the top and internal margin of the socket. The form of the acromion process was changed; the capsule smaller than natural, the head of the humerus irregularly oval, its anterior half alone being in contact with the glenoid cavity; the greater tubercle natural, but the lesser elongated and curved, forming a process of an inch in length, around the base of which the tendon of the biceps muscles played."*

In cases where the humerus has accommodated itself to its new position, as in the foregoing, any attempt at reduction would doubtless prove futile, and might be followed by disastrous consequences. In a case reported by Gaillard, he succeeded in reducing a dislocation in a young lady, aged sixteen, which had existed from birth. The head of the bone rested in the infra-spinous fossa; the scapula, clavicle and arm, were preternaturally small. The arm was made to undergo a preparatory course of extension and manipulation. On the fourth attempt it slipped into its socket, but at once became re-dislocated. By means of bandages it was retained in place on a second attempt, and at the end of two years had so much increased in strength, size and usefulness, as to encourage the hope that a cure would result.†

From the nature of the case, it is evident that the earlier the treatment is commenced, the greater probability there will be of success. In young infants, there will generally be but little difficulty in restoring the head of the bone to its place; but from certain anomalies in the attachments of the muscles, and from the extensive rupture of the capsular ligament incident to the attempts at reduction, great difficulty will be experienced in retaining the bone in place.

The following case of congenital dislocation, in an infant of eleven months, while it presents nothing out of the general line, will be found, perhaps, as an illustration of the value of the method adopted in its reduction, and as illustrating the possibility of reduction in similar cases, to present points of interest and value.

On the sixteenth of December, 1872, Mrs. F——, of Wellsville, Allegany Co., N. Y., brought her infant son Eddie to Dr. Miner on account of some trouble with his left arm and shoulder. The child was eleven months old, was well developed in every respect, was healthy, and well nourished. He had, from birth, exhibited a reluctance to move his left arm, and manifested evidences of pain when it was handled. Physicians who had been consulted had given

*R. W. Smith quoted by Hamilton. Fractures and Dislocations—p. 769.
†Gaillard Mem. de l'Acad. de Med., 1841. From Hamilton, 1871.

different views upon the subject, generally being of the opinion that the child would outgrow the difficulty. The child had never received any blow or fall that could account for the lameness, neither had the mother been injured in any way during pregnancy. At her confinement she was attended by a midwife, who used some violence in the delivery. She did not know what the presentation was, but thinks, from what her attendant told her, that it was not the head. Taking these facts into consideration, it is probable that the injury occured during delivery, and was not an accident of intra-uterine life. We may apply to it, however, the term congenital, as it certainly existed from birth.

An examination of the arm showed that motion could be pretty freely made from before backward, but that movements of the arm outward caused pain. There was a depression beneath the acromion process, of considerable depth, and a round body could be felt occupying the axilla. The elbow was carried somewhat backward, and away from the thorax. When the hand was placed over the opposite shoulder, the elbow projected still further from the side. A diagnosis of dislocation into the axilla was easily made, and from the general mobility of the joint, a favorable prognosis given.

December 18, the child was placed under the influence of chloroform, and the reduction made in the following manner: The scapula was firmly fixed in position by the writer. Grasping the arm with his right hand, and placing his left thumb in the axilla to guide the head of the bone, Dr. Miner attempted reduction by the usual mode of extension. This failing, after repeated trials, was discontinued. Substituting his left hand by that of his assistant, he flexed the forearm upon the arm, and making extension from above the elbow, the arm was carried outward from the body at nearly a right angle; then, using the forearm as a lever, the arm was rotated outward to its extreme limit, the head of the bone being at the same time forced upward by the assistant. This procedure was successful in restoring the head of the bone to the glenoid cavity, in which, as it rotated, it produced a fine, almost crepitant, sensation, showing that a deposit had formed in the socket. The elbow was supported in a sling, and the arm retained in position by broad bands of adhesive plaster and bandages. But little inflammatory action was produced by the operation, and in a short time the child was discharged with a perfectly restored shoulder.

REMARKS.—The treatment employed in this case, and all those referred to in this article, will be seen to be a modification, in some respects, of the method of manipulation introduced by Prof. H. H. Smith, of Philadelphia. The resemblance lies in the rotation of the arm outward, while at an angle with the body, using the flexed forearm as a lever. Prof. Smith proceeds further than this, and in recent cases, his method is often attended with success. Having rotated the arm upward and outward, he reverses the movement by rotating the head downward and inward, at the same time carrying the elbow to the side. In ancient dislocations, however, this method of manipulation alone, will not be found to succeed. The modification consists in employing some force to act upon the head of the bone, while the extension and outward rotation is being made. This may be accomplished by the heel in the axilla, or by a counter-extending band passed around the upper portion of the humerus. The use of the heel is probably more free from the danger of the fracture of the hnmerus, but in either case this accident will rarely happen, where a proper amount of force is employed, except in old persons. If the counter-extending band is employed, the assistant should be instructed to make traction in a steady manner, and should thoroughly understand the object of the manipulation so as to change the line of traction at the proper moment. As the arm is carried out from the body, it may be gently rocked back and forth, to assist in breaking up adhesions, at the same time this manipulation may cause it to enter more readily into the rent in the capsule. It is, however, at the moment of extreme rotation outward, that the reduction will be found more often to take place. M. Revillout gives an account in the *Gazette des Hopitaux* of July 31,* of the mode employed by M. Panas, in the reduction of dislocations of the head of the bone forward. M. Panas believes that almost all these dislocations are produced by a rotation of the head of the humerus, and as the result of experiments, he has been able to demonstrate that by a movement of

*American Journal of Medical Science, October, 1875, p. 551.

rotation it is easy to lacerate a capsular ligament, which would resist a direct traction of six hundred kilogrammes. Once beyond the laceration of the capsule, the head of the bone, when carried inwards, lies supported on the inner lip of the laceration, and if reduction be attempted in this position, it is necessary to rupture the more or less broad ligamentous bridle which separates it from the glenoid cavity. When, however, the muscular resistance has been overcome by a sufficient extension, the rotation outwards brings the head to the middle of the rupture, and a slight movement suffices to throw it into place. This mode of M. Panas resembles almost exactly that employed by Dr. Miner in the reduction of the cases of sub-glenoid dislocation referred to in this paper. In a recent case, complicated by fracture of the forearm, the writer succeeded in restoring without difficulty a dislocation into the axilla where the employment of the heel in the axilla could not, from the nature of other injuries, be brought into requisition. Resembling, in its general principle, the methods of manipulation described by Dr. H. H. Smith, and others, its simplicity, and ease of application, makes this method worthy of trial by the profession.

